# Comparing pre- and post-copulatory mate competition using social network analysis in wild crickets

**DOI:** 10.1093/beheco/arv236

**Published:** 2016-01-10

**Authors:** David N. Fisher, Rolando Rodríguez-Muñoz, Tom Tregenza

**Affiliations:** Centre for Ecology and Conservation, University of Exeter, Penryn Campus, Treliever Road, Penryn, Cornwall TR109FE, UK

**Keywords:** cryptic female choice, *Gryllus*, male competition, sexual selection, sperm competition.

## Abstract

Sexual selection results from variation in success at multiple stages in the mating process, including competition before and after mating. The relationship between these forms of competition, such as whether they trade-off or reinforce one another, influences the role of sexual selection in evolution. However, the relationship between these 2 forms of competition is rarely quantified in the wild. We used video cameras to observe competition among male field crickets and their matings in the wild. We characterized pre- and post-copulatory competition as 2 networks of competing individuals. Social network analysis then allowed us to determine 1) the effectiveness of precopulatory competition for avoiding postcopulatory competition, 2) the potential for divergent mating strategies, and 3) whether increased postcopulatory competition reduces the apparent reproductive benefits of male promiscuity. We found 1) limited effectiveness of precopulatory competition for avoiding postcopulatory competition; 2) males do not specifically engage in only 1 type of competition; and 3) promiscuous individuals tend to mate with each other, which will tend to reduce variance in reproductive success in the population and highlights the trade-off inherent in mate guarding. Our results provide novel insights into the works of sexual competition in the wild. Furthermore, our study demonstrates the utility of using network analyses to study competitive interactions, even in species lacking obvious social structure.

## Lay Summary

In many animals, males compete for fertilizations both before and after mating. But do males specialize in 1 type of competition? And do physical fights between males lead to less competition between their ejaculates within females? We studied competitions between wild crickets by building networks of interactions. We found that males that had more fights were more likely to meet in sperm competition, suggesting that evolution will not favor specialists in one of the 2 types of competition.

Twitter: @DFofFreedom

## INTRODUCTION

Competition for mates has a potent influence on evolution. Females may prefer particular males and dominant individuals can monopolize access to females (Birkhead and Pizzari 2002; Andersson and Simmons 2006), leading to variance in fitness that drives selection. Additionally, in both internally and externally fertilizing species, once matings are achieved there is still room for further sexual selection through processes such as sperm competition (Parker 1970) and cryptic female choice (Thornhill 1983). This divides sexual selection into 2 arenas of competition: precopulatory and postcopulatory (“episodes of selection” according to Pizzari et al. 2002). These arenas of competition are however not necessarily independent (Yasui 1997; Tomkins et al. 2004). A number of studies have identified negative associations across species between sexual dimorphism in body size (an indicator of males’ ability to monopolize access to females) and relative testes size (an indicator of the strength of postcopulatory selection) (Heske and Ostfeld 1990; Poulin and Morand 2000). This pattern is consistent with the hypothesis that intense precopulatory competition leads to reduced postcopulatory competition between individuals. Furthermore, ability in pre- and post-copulatory competition within individuals can be positively related (e.g., Matthews et al. 1997; Hosken et al. 2008), negatively related (e.g., Pizzari et al. 2002; Simmons and Emlen 2006; Demary and Lewis 2007; Engqvist 2011), or show no relationship (e.g., Lewis et al. 2013; reviewed by Mautz et al. 2013). If ability in pre- and post-copulatory competition is negatively related within individuals, then divergent male morphs specializing in either mode of competition, such as those found in the beetle *Onthophagus binodis* (Cook 1990), can evolve. If there is a genetic correlation between ability in the 2 types of competition, the rate of evolutionary change in traits will be increased if the correlation is positive and retarded if it is negative (Andersson and Simmons 2006; Mautz et al. 2013).

If males with many matings are disproportionately more likely to mate with females who also have a higher than average numbers of partners, their reproductive success will be reduced due to a loss of paternity through sperm competition (Sih et al. 2009). This will lower the variance in reproductive success in the population and weaken precopulatory selection (e.g., Danielsson 2001). The relationship between mating rate and reproductive success could even be completely reversed if the positive association between male and female mating rate is strong enough (McDonald and Pizzari 2014). Alternatively, if males who mate frequently also achieve high exclusivity, then the variance in reproductive success will instead increase. Therefore, the potentially major implications for fitness and evolution of both these arenas of competition make understanding the relationship between them important (Preston et al. 2003; Hunt et al. 2009; Sbilordo and Martin 2014).

In the field cricket *Gryllus campestris* individuals live in and around burrows, they dig as nymphs in the autumn and continually enlarge as they grow. *Gryllus campestris* will only share their burrow with a member of the opposite sex once they are adult and fights occur intrasexually in both males and females. Male–male fights are assumed to be contests for access to mating partners (Alexander 1961; Rodríguez-Muñoz et al. 2011). Both sexes seek out multiple mates (Rodríguez-Muñoz et al. 2010), so males are frequently in sperm competition (Tyler et al. 2013). This mating system, with its high levels of both pre- and post-copulatory competition provides an opportunity to study the relationship between these types of male–male competition. We tested the following 3 sets of predictions:

1) Different patterns of dominance could lead to different relationships between pre- and post-copulatory competition between males. If dominant males prevent others from mating by evicting them from burrows, within a pair of crickets, there would be a negative relationship between the intensity of pre- and post-copulatory competition. Alternatively, males may fight more when they are of a similar fighting ability, and so a clear dominance hierarchy cannot be established. In such a situation, a female may not be able to choose between them and so mate with them both. This would result in positive associations within a pair for the intensity of the 2 types of competition.
2) If some individuals can consistently evict others from burrows, this could then lead to individuals specializing in either pre- or post-copulatory competition. This would result in negative relationships within individuals between engagement in pre- and post-copulatory competition. However, crickets are thought to possess flexible mating strategies (Buzatto et al. 2014), so we do not expect individuals to consistently trade-off between the 2 types of competition. Instead, positive relationships based on condition or quality seem more likely (e.g., Hosken et al. 2008).
3) Although males may attempt to dominate one another to maximize their reproductive success, females may mate multiply with both dominant and nondominant males. This would tend to reduce the success of dominant males through sperm competition, resulting in a reduction in the variance in reproductive success and precopulatory selection within a population (Sih et al. 2009). The use of mating success as a proxy for reproductive success could then lead to misleading results. Alternatively, males who are successful at acquiring matings may also be successful at preventing females from remating, which would have the opposite effect.

Directly comparing pre- and post-copulatory competition to investigate these predictions is a challenge. Although pre- and post-copulatory competition differ in how the individuals interact, the time and spatial scale they interact at and the currency of victory, they can both be represented in the same way: as a social network. Links (“edges” in network terminology) can be drawn between individuals (“nodes”) if they engage in pre- or post-copulatory competition with each other (McDonald et al. 2013), representing the population as a network. For example, 2 males can be linked if they fight one another for access to a female or they can be linked by both mating with the same female within a timeframe that places them in sperm competition with one another. Alternatively, an entire mating system can be represented as a network of male–female links. This network approach allows the researcher to quantify each individual’s unique social and competitive environment, thus providing more accurate estimates of the selection that a population is under (McDonald et al. 2013).

The networks can be analyzed using social network analysis (SNA). For instance, the centrality measure “degree,” which is the number of unique edges a node possesses, can be used to quantify the intensity of competition an individual is experiencing (McDonald et al. 2013). Recent reviews have highlighted that SNA can be used even with animals not typically considered “social” (Krause et al. 2009; Sih et al. 2009; Krause et al. 2011; McDonald et al. 2013; Pinter-Wollman et al. 2013; McDonald and Pizzari 2014; Kurvers et al. 2014). For example, Muniz et al. (2014) examined differences between territorial and sneaker males using social networks of male harvestmen (*Serracutisoma proximum*). They found that territorial males experienced less sperm competition on average, but the amount was more variable than for sneaker males. Meanwhile, in mating networks of the Asian red palm weevil (*Rhynchophorus ferrugineus*) males show greater variance in the number of unique individuals they interact with than females, suggesting that males are under stronger sexual selection (Inghilesi et al. 2015).

To build on these applications of SNA, we observed the interactions and movements among burrows of a wild population of field crickets (*G. campestris*). Burrows are dug by nymphs in the autumn and are used to avoid predators and adverse weather conditions. *Gryllus campestris* is univoltine, and adults are active between April and July. Sexual activity begins 2–5 days after the final molt to adulthood when males start to sing from their burrows to attract mates and both sexes move among burrows in search of mates. Observing males fighting each other, and mating with the same female allows us to construct networks of pre- and post-copulatory competition in order to test the 3 sets of predictions presented above.

## METHODS

### Data collection

Observations were made at the “WildCrickets” project site in Northern Spain (Rodríguez-Muñoz et al. 2010). Each spring since 2006, we have located and marked each burrow at our study site. Around mid-April, to coincide with when adults start to emerge, we placed cameras over burrows with nymphs, allowing us to record the emergence dates and subsequent behavior of adult crickets. We have completed video analysis of cricket interactions for the years 2006 and 2013, and these are the data used in this study. There were 64 cameras in 2006 and 120 in 2013, and the total adult population sizes were 151 and 239, respectively. In both years, at the peak of the season, there were more burrows than cameras, so we moved cameras from burrows that had recorded no activity for 2–3 days to monitor as many individuals as possible. In 2006, individuals were observed for a mean of 11.8±10.7 days (mean ± standard deviation), and in 2013, they were observed for 13.6±10.1 days. The majority of behavior related to reproductive success takes place at the burrows (Rodríguez-Muñoz et al. 2010). We also directly observed burrows without a camera, to assess when the resident nymph became an adult. Late instar nymphs rarely move among burrows, so we can be confident that we correctly assigned emergence dates to most of the population.

Three or 4 days after each cricket emerged as an adult, we trapped it and glued to its thorax a unique visible tag with a 1 or 2 character codes. This allowed individual identification without disrupting natural behavior. Following this tagging process, we released the cricket back to the burrow we trapped it from. The burrow was blocked while the cricket was being tagged to prevent other animals, including other crickets, from usurping the burrow. We then watched the video recordings to record cricket behavior such as movement among burrows, mating, fighting, and predation events for each individual until its death. When the death of a cricket was not directly observed, we assigned the last observation date as the date of death. Migration in and out of the study area is relatively low (Rodríguez-Muñoz et al. 2010), so we are confident that if a cricket is no longer observed it has died rather than moved to a new area.

### Network construction

We constructed 2 networks, each representing a type of male–male competition:

1) Fighting. We linked one male to another if it arrived at a burrow and fought the resident. This ranges from flaring mandibles to wrestling (Alexander 1961). The strength of the interaction was a count of the number of times the cricket arrived at a burrow and fought a particular opponent. This network was therefore weighted and asymmetrical (directed).
2) Sperm competition. Insects store sperm in their spermatheca, and multiple paternity has been demonstrated in field crickets, so males mating to the same female will be in sperm competition (Bretman and Tregenza 2005; Tyler et al. 2013). Using only matings where a spermatophore was successfully transferred, we created a network of mating between males and females. This is distinct from a typical network in that the matrix is rectangular and links only exist between 2 types of individuals, never between 2 of the same type of individual (a bipartite network). We then linked males if they mated with the same female. Two males were interacting more strongly with each other if they mated many times with the same females. A male interacted with another with equal strength if they both mated once with 2 females or if they each mated twice with a single female. Our rationale being that each spermatophore represents a unit of investment by the male, competing for a share of the female’s eggs. In both situations, ignoring order effects and assuming equal competitive ability, a male has equal chance of fertilizing each available egg, regardless of whether they are split across 2 females or 1. This network was asymmetrical, with one male having a link to another male equal to the total number of times the first male mated with any female also mated by the second male, and vice versa.

### Similarity in space and time

Two individuals are likely to be in greater pre- and post-copulatory competition if they overlap in space and time compared with a pair that did not. To account for this, we constructed matrices of individuals’ temporal and spatial overlap during their adult lives. The former was simply the number of days that each pair of adult crickets were alive at the same time, a symmetrical relationship. For spatial overlap, we linked males via their interactions with burrows. Encounters between individuals away from burrows are likely to be very rare because individuals spend the vast majority of their time in the immediate vicinity of burrows, typically leaving only when moving to another burrow. Each male’s strength of interaction with a particular burrow was equivalent to the amount of time he was observed there. Males were then connected to other males who also used each particular burrow. These edge weights, and so the matrix, was initially asymmetrical, as each male using a burrow would have spent different amounts of time there. To obtain a single value for each pair that represented how close in space they were, the matrix was symmetrized by taking the geometric means of the 2 values. For pairs of values, the geometric mean is the square root of their product. This effectively gives greater weight to values close together rather than those further apart. For example, the geometric mean of 5 and 5 is 5, but the geometric mean of 1 and 9 is 3. Both of these pairs would have an arithmetic mean of 5, but we do not think they represent equal strengths of interaction, which the use of the geometric mean captures more accurately. Two males that spent longer at the same set of burrows as each other (but not necessarily at the same time) were more strongly connected.

### Network analyses

For prediction (1), about relationships between the intensity of pre- and post-copulatory competition within pairs of competing males, we used an extension of quantitative assignment procedure to multiple regression: MRQAP (Krackhardt 1988). We used an ordinary least squares (OLS) network regression with the sperm competition network as a response variable and the network of fighting as a predictor variable. We controlled for a pair’s similarity in time and space by adding the matrices for spatial and temporal overlap as covariates. To independently estimate the effect of each of the predictor variables on the response variable, the test was performed using Dekker semipartialling for the permutation tests (see Dekker et al. 2007) in the R package “sna” (Butts 2008). This was necessary as the covariates were significantly correlated (Mantel tests, 2006: *r* = 0.354–0.544; 2013: *r* = 0.184–0.442, *P* < 0.001 in all cases). We symmetrized the fighting and sperm competition networks, to give a single value for an edge between 2 individuals indicating how strongly they were linked in competitive interactions, rather than how much one interacted with another. This was done by taking the geometric mean of the 2 edge weights as for the spatial closeness network. This allows us to determine whether the level of sperm competition within each pair was positively, negatively, or not associated with the frequency of fighting within the pair. For each predictor variable, we subtracted the mean pairwise interaction strength from each value to center the values over zero and divided by the standard deviation of all pairwise interaction strengths. This means that each variable was on the same scale (number of standard deviations the datum is above/below the mean), which aids interpretation (Hunt et al. 2005; Schielzeth 2010).

For prediction (2), about the correlation within individuals in engagement in pre- and post-copulatory competition, we correlated an individual’s degree between each of the networks. We repeated this using individual “strength,” that is, the total number of interactions an individual instigated, regardless of who they were with. This is distinct from the previous analysis, which compares the within-pair relationships between networks, and used the original, directed/asymmetric networks. Therefore, to test predictions (1) and (2), we looked at both within-pair and within-individual relationships between pre- and post-copulatory competition.

For prediction (3), if promiscuous males mate with promiscuous females, we took each connection in the male–female mating network and correlated the degrees of the individuals at either end. This measure is known as “degree correlation”; a positive correlation indicates that individuals with many connections are connected to other individuals with many connections, whereas a negative correlation indicates that individuals with many connections are primarily connected to individuals who are connected to few others (Newman 2002, 2003). We compared the observed correlation with the correlation found in 1000 simulated networks. For these networks, we first multiplied together the spatial and temporal overlap matrices, to create a network that only contained nonzero values for pairs of crickets that were both alive at some point and were observed to use the same burrow at least once. This represented all possible connections. We then took a random subsample of the edges in this network 1000 times, to give 1000 random networks with, on average, the same density as the observed network. This accounted for nonzero degree correlations that could arise through spatial and temporal structuring. *P* values were obtained as the proportion of simulated values with more extreme correlations than the observed network (Simpson 2015).

## RESULTS

There were 74 males in 2006 and 119 in 2013. In 2006, there were 35 males that never used the same burrow as another male, and 23 such males in 2013. These isolated individuals were not considered for the analyses of interactions, as they could not contribute to sexual selection through fighting and were unlikely to contribute through sperm competition. The frequency of these individuals was higher in 2006 than 2013 (35/74 and 23/119, respectively). Individuals were observed for a similar mean amount of time in each year, so this difference presumably reflects the lower population density in 2006.

**Figure 1:**
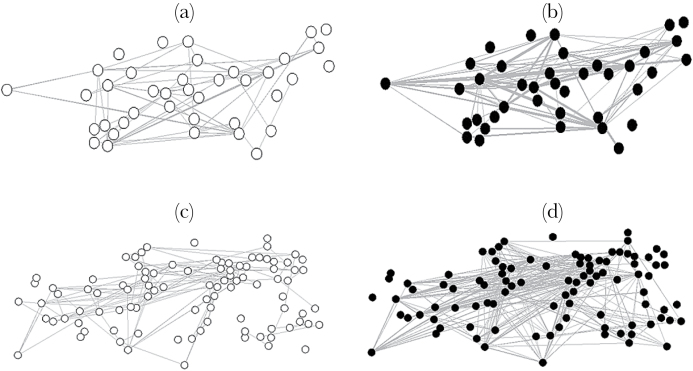
Plots of the networks in 2006 (a and b) and 2013 (c and d). The networks of fighting are plotted with open circles (a and c), and the sperm competition network with solid circles (b and d). Lines indicate males that either fought each other (fighting) or mated with the same female (sperm competition). Each male is plotted in the same position in each network, based on his emergence location as an adult, so individuals occupy the same position in the fighting and sperm competition networks.

Not every male necessarily interacted in every network; for instance, if they fought, but never successfully mated with a female, they would score zeros for sperm competition with all other males but would still be included in the analyses. Plots of each network are shown in Figure 1a–d. Each male possessed a similar degree in the fighting network as the sperm competition network in 2006 (medians of 1 and 2, respectively, Wilcoxon test, *W* = 633, *N* = 39, *P* = 0.194), but males had a higher degree in the sperm competition network in 2013 (medians of 1 and 2.5 for the fighting and sperm competition, respectively, Wilcoxon test, *W* = 3280, *N* = 96, *P* < 0.001).

### Within-pair intensity of pre- and post-copulatory competition

The results of the OLS network regression are presented in Table 1. In both years, the networks of fighting and the matrices of spatial and temporal overlap were significant, positive predictors of the networks of sperm competition.

**Table 1:** Results of OLS network regression for the effect of fighting frequency, spatial similarity, and overlap in lifespan on the level of sperm competition between males

Year	Predictor coefficients		*P*	Model statistics	
2006	Fighting	0.446	0.002	Residual standard error	2.132
	Space	1.16	<0.001	Degrees of freedom	737
	Time	0.374	0.003	*R* ^2^	0.385
2013	Fighting	0.186	<0.001	Residual standard error	0.824
	Space	0.215	<0.001	Degrees of freedom	4556
	Time	0.134	<0.001	*R* ^2^	0.187

Each of the predictor variables has been mean centered and scaled to unit variance, so effect sizes are comparable.

### Within-individual correlation between engagement in pre- and post-copulatory competition

**Figure 2:**
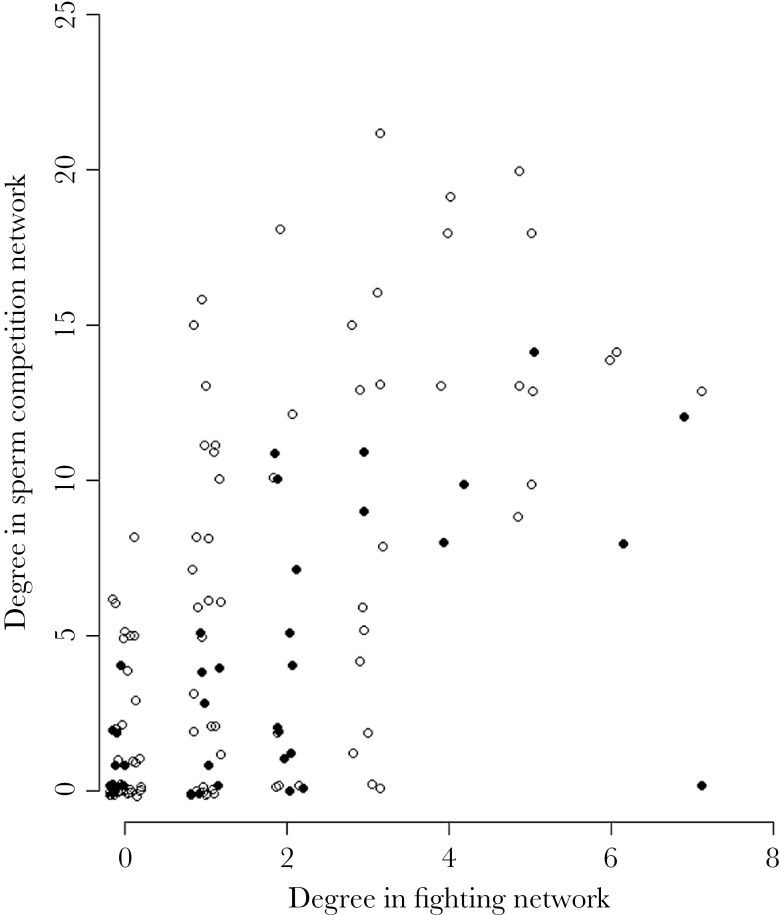
Each male’s degree in the fighting network against his degree in the sperm competition network. Filled circles = 2006, open circles = 2013. There were strong positive correlations in both years (2006: *r*_s_ = 0.594; 2013: *r*_s_ = 0.576). A small value has been added to each point at random to reveal that there are multiple points for some *x* and *y* values.

An individual’s degree in the fighting network was positively correlated with its degree in the sperm competition network (Figure 2; Spearman rank correlation, 2006: *N* = 39, *S* = 4000, *r*_s_ = 0.595, *P* < 0.001; 2013: *N* = 96, *S* = 62500, *r*_s_ = 0.576, *P* < 0.001). This result was maintained if an individual’s strength was used in place of degree (2006: *N* = 39, *S* = 3640, *r*_s_ = 0.631, *P* < 0.001; 2013: *N* = 96, *S* = 64500, *r*_s_ = 0.563, *P* < 0.001).

### Promiscuous crickets tend to mate with each other

**Figure 3:**
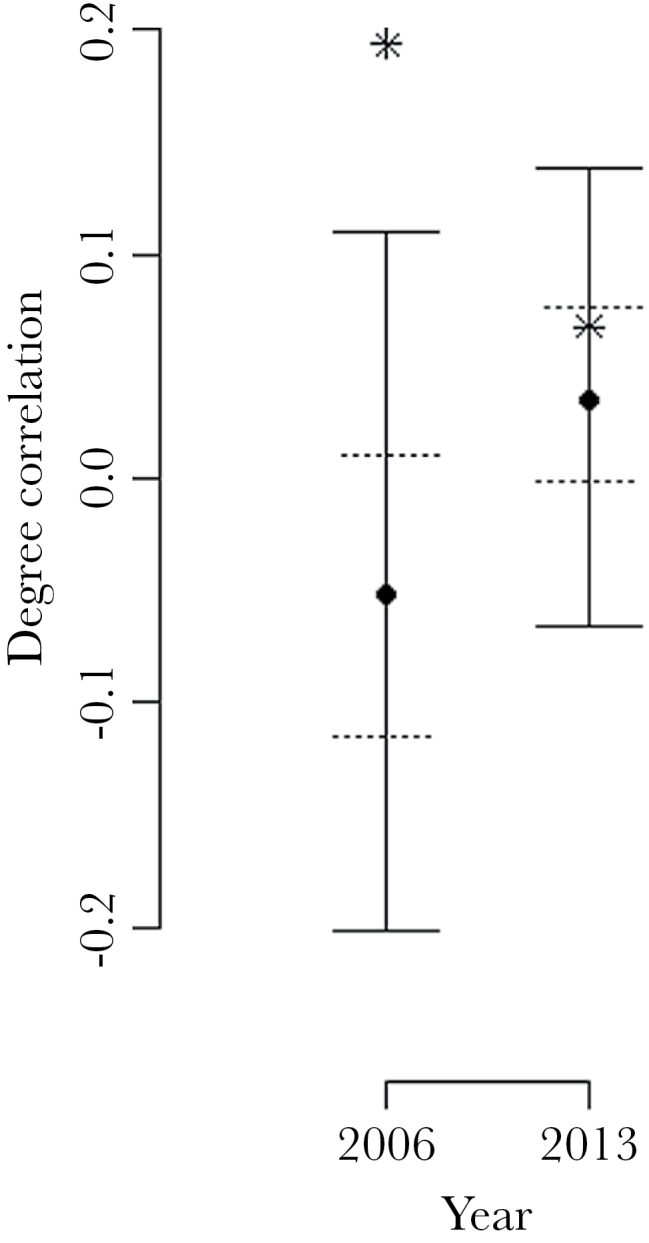
Plots of the simulated and actual correlations between male degree and female degree from the mating network in each year. The solid points indicate the medians, dashed horizontal lines the 50% quantiles, and the solid horizontal lines the 95% quantiles. Simulated networks possessed only links between crickets that overlapped in both space and time, and were on average the same density as the original network (see Methods for details). The observed value for each year is plotted as an asterisk. The correlation in 2006 (0.193) was greater than 99.7% of simulated correlations, whereas the correlation in 2013 (0.068) was greater than 70% of simulated correlations.

There was a positive degree correlation in the male–female mating network in 2006, but there was no correlation in 2013 (Spearman rank correlation, 2006: *N* = 93, *r*_s_ = 0.193, permutation *P* value = 0.003; 2013: *N* = 246, *r*_s_ = 0.068, permutation *P* value = 0.300). Plots of the simulated versus observed correlations are shown in Figure 3.

## DISCUSSION

We found that 1) males were in stronger sperm competition with the males they fought more; 2) males that fought with many different males were also in sperm competition with many different males; and 3) there is a positive relationship between the promiscuity of a male and the promiscuity of the females he mated with but it is only statistically significant in 2006. We address each of these findings in turn.

### Within-pair and within-individual correlations between pre- and post-copulatory competition

Males that emerged as adults nearer each other engaged in more intense sperm competition. Similar results have been found in harvestmen (*S. proximum*), where harems of females that were close together were more likely to be invaded by the same “sneaker” male (Muniz et al. 2014), whereas trees (*Prunus mahaleb*) near each other tend to be visited by the same pollinators (Fortuna et al. 2008). The temporal overlap of male ejaculates is necessary for sperm competition to occur (Wigby and Chapman 2004), and individuals typically reduce competition by segregating themselves in time (e.g., Alanärä et al. 2001). However, we are unaware of any studies in wild animals that explicitly demonstrate that the degree of spatial and temporal overlap of adult males increases the intensity of sperm competition between them. It seems probable that this is a pattern that is likely to be a general feature of spatially structured populations. This finding highlights how decisions made by mothers over factors such as egg laying site or nest location (Refsnider and Janzen 2010; Mitchell et al. 2013) or aspects of phenology such as laying date (Einum and Fleming 2000; Skoglund et al. 2011) can strongly influence the competitive environment of offspring.

Even after accounting for temporal and spatial factors, the number of fights between a pair of males was positively related to their level of sperm competition. This suggests that precopulatory competition may not always be an effective means of avoiding postcopulatory competition. This is true in various systems, for instance some male giant cuttlefish (*Sepia apama*) change markings to look like females, allowing them to mate with females guarded by larger males (Norman et al. 1999), whereas female brown capuchin monkeys (*Sapajus apella*, formerly *Cebus apella*) solicit copulations from subordinate males toward the end of their estrous cycles (Janson 1984). As this relationship between pre- and post-copulatory competition does not appear to be explained by patterns of space use or phenological equivalence, some other factor must be driving males in one form of competition to be more involved in the other type. One possible explanation is that there is some consistency among females in their preference for males of a particular type. Males of this type will be more likely to be in sperm competition with one another because they receive a disproportionate share of matings. If males that are similar in their attractiveness to females are also more similar in their fighting abilities, then they may be evenly matched in fights, which may lead to them having repeated fights with one another as they seek to establish dominance. The 2 perquisites for this possibility have been established in other animals. Consistent female preferences for certain male types have been shown in a number of species in laboratory settings (Howard and Young 1998; Forstmeier and Birkhead 2004; Cummings and Mollaghan 2006). Furthermore, escalation of fights between male butterflies typically occurs when each male considers himself to be the resident of the territory, a role that normally settles contests (reviewed in Kemp and Wiklund 2001). Similarly, prolonged contests between female house finches (*Carpodacus mexicanus*) only occur between females most closely matched in condition and body size (Jonart et al. 2007). Therefore, fighting behavior may be an emergent property of equality in sexual competition, rather than a means of imposing inequality.

Our observation of a positive correlation in degree within individuals between networks indicates that individuals that instigated many fights were also engaged in a lot of sperm competition. This may be related to our earlier observation that pairs of males in premating competition were more likely to be in postmating competition, which we interpreted as likely being the result of closely matched males tending to fight frequently, and also being unable to exclude one another from females and hence experiencing high sperm competition. This within-pair correlation could drive within-male correlations in pre- and post-copulatory competition because males that happen to encounter an opponent of similar competitive ability will experience a lot of fights and a lot of postcopulatory competition, whereas males that only encounter opponents of divergent fighting ability will tend to have fewer fights and experience less sperm competition. The positive correlation between degrees in each network could potentially amplify the reproductive success of good condition or high-quality males if abilities in pre- and post-copulatory forms of competition are correlated through links to condition or quality (e.g., Matthews et al. 1997; Hosken et al. 2008). There is a large reproductive skew observed among males in this population (Rodríguez-Muñoz et al. 2010), suggesting that this may be occurring. This type of skew in mating success is common in social animals due to control of mating opportunities by dominants or strong benefits of kin-directed altruism (Engh 2002; Hager and Jones 2009; Ryder et al. 2009; Cant et al. 2010). This skew in crickets and other nonsocial animals could be driven by differences in longevity, as the number of fights instigated and copulations achieved is expected to increase over time. Together, these results suggest that males must be adapted to both pre- and post-copulatory competition, as they will typically be engaged in both. This therefore makes the evolution of alternative male morphs unlikely. As crickets do not have a particularly unusual mating system, it seems likely that this correlation will be typical of species where the potential for males to monopolize females or resources is limited (Andersson and Simmons 2006; Buzatto et al. 2014).

### Promiscuous crickets mate with each other

Promiscuous individuals positively assorted in both years although it was not significant at the 95% level in 2013. Therefore, in 2006 at least, apparently successful males faced higher sperm competition for each of their matings than did less promiscuous males. This would have tended to reduce the variance in reproductive success across the population (Sih et al. 2009). This could result from positive assortment by attractiveness due to both females and males exercising choice of mates. Such mutual mate choice has been found in a number of animals, for example, in a cichlid fish (*Pelvicachromis taeniatus*), large body size was favored by both sexes but larger individuals were more choosy, resulting in positive assortment by body size (Baldauf et al. 2009). Furthermore, the strength of sexual selection acting on male and female fruit flies (*Drosophila serrata*) has been shown to be approximately equal, with a low genetic correlation suggesting independent evolution of sexually selected cuticular hyrdocarbon profiles (Chenoweth and Blows 2003). Therefore, males who are preferred by females may also prefer particular females, who are attractive to all males. This would result in the individuals with the highest mating rates mating with each other; the pattern we observe here. This positive correlation between mating rates also indicates that males with many mating partners are less successful at preventing females from remating than those who only mate with a few different females. This trade-off between the mating rate of a male and the fidelity of his partners may explain the considerable variance in mate-guarding behavior in this population (Rodríguez-Muñoz et al. 2011). Our results support the argument that, in systems with frequent postcopulatory competition, using the number of matings as a proxy for reproductive success may lead to the overestimation of the fitness of males who mate often (Preston et al. 2001). This should encourage more studies into the fitness determinants of polyandrous species in the wild (Preston et al. 2003; Rodríguez-Muñoz et al. 2010; McFarlane et al. 2011; Thompson et al. 2011; Sardell et al. 2012).

## CONCLUSIONS

Following recent calls (McDonald et al. 2013; McDonald and Pizzari 2014), we used methods of data analysis not commonly used in the field of sexual selection to provide insights into male–male competition in a wild population. By simultaneously considering pre- and post-copulatory sexual competition among individuals of an entire population, as well as both individual and pairwise relationships between the 2 types of competition, we have addressed a range of questions relevant to promiscuous mating systems. We found that males are unlikely to specialize in either pre- or post-copulatory competition nor can they use the former to avoid the latter. This supports the idea that in species where males are unable to monopolize access to females the evolution of alternative male phenotypes is unlikely. Furthermore, the structure of the mating network may reduce variance in reproductive success, reducing the usefulness of mating success as a proxy for reproductive success, and suggests that males who mate more often may lose more paternity through sperm competition.

## FUNDING

Funding for this research was provided by NERC (studentship no.: NE/H02249X/1; grant no.: NE/H02364X/1). Further support was provided by the University of Exeter’s Postgraduate Research Enhancement Fund, awarded to D.N.F.
